# Redetermination of (*E*)-3-(anthracen-9-yl)-1-(2-hy­droxy­phen­yl)prop-2-en-1-one^1^

**DOI:** 10.1107/S1600536811034994

**Published:** 2011-09-03

**Authors:** Suchada Chantrapromma, Thawanrat Kobkeatthawin, Kullapa Chanawanno, Jaruwan Joothamongkhon, Hoong-Kun Fun

**Affiliations:** a Crystal Materials Research Unit, Department of Chemistry, Faculty of Science, Prince of Songkla University, Hat-Yai, Songkhla 90112, Thailand; b X-ray Crystallography Unit, School of Physics, Universiti Sains Malaysia, 11800 USM, Penang, Malaysia

## Abstract

The redetermined structure of title chalcone derivative, C_23_H_16_O_2_, corrects errors in the title, scheme and synthesis in the previous report of the same structure [Jasinski *et al.* (2011 ▶). *Acta Cryst.* E**67**, o795]. There are two independent mol­ecules in the asymmetric unit with slight differences in bond lengths and angles. The dihedral angle between the benzene ring and the anthracene ring system is 73.30 (4)° in one mol­ecule and 73.18 (4)° in the other. Both mol­ecules feature an intra­molecular O—H⋯O hydrogen bond, which generates an *S*(6) ring. In the crystal, mol­ecules are arranged into sheets lying parallel to the *ac* plane and further stacked along the *b* axis by π–π inter­actions with centroid–centroid distances in the range 3.6421 (6)–3.7607 (6) Å. The crystal structure is further stabilized by C—H⋯π inter­actions. There are also C⋯O [3.2159 (15) Å] short contacts.

## Related literature

For the previous structure determination, see: Jasinski *et al.* (2011 ▶). For a related structure and background references, see: Joothamongkhon *et al.* (2010 ▶). For graph-set motifs, see: Bernstein *et al.* (1995 ▶). For the stability of the temperature controller used in the data collection, see Cosier & Glazer (1986 ▶).
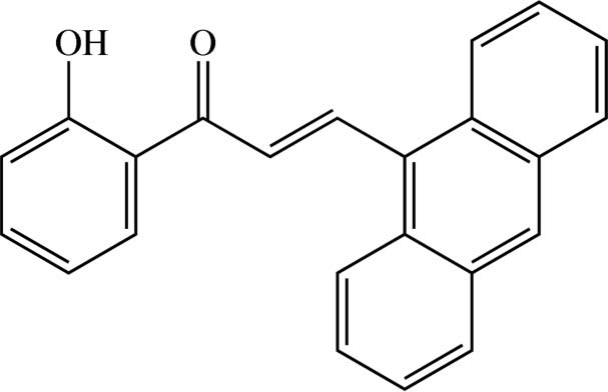


## Experimental

### 

#### Crystal data

C_23_H_16_O_2_
*M* *_r_* = 324.36
Monoclinic, 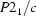

*a* = 14.0843 (2) Å
*b* = 13.7224 (2) Å
*c* = 16.9615 (3) Å
β = 101.411 (1)°
*V* = 3213.36 (9) Å^3^
*Z* = 8
Mo *K*α radiation
μ = 0.09 mm^−1^
*T* = 100 K
0.50 × 0.39 × 0.37 mm

#### Data collection

Bruker APEXII CCD diffractometer
Absorption correction: multi-scan (*SADABS*; Bruker, 2005 ▶) *T*_min_ = 0.959, *T*_max_ = 0.969
40230 measured reflections
9368 independent reflections
7868 reflections with *I* > 2σ(*I*)
*R*_int_ = 0.032

#### Refinement

*R*[*F* ^2^ > 2σ(*F* ^2^)] = 0.046
*wR*(*F* ^2^) = 0.132
*S* = 1.02
9368 reflections
459 parameters
H atoms treated by a mixture of independent and constrained refinement
Δρ_max_ = 0.54 e Å^−3^
Δρ_min_ = −0.21 e Å^−3^

### 

Data collection: *APEX2* (Bruker, 2005 ▶); cell refinement: *SAINT* (Bruker, 2005 ▶); data reduction: *SAINT*; program(s) used to solve structure: *SHELXTL* (Sheldrick, 2008 ▶); program(s) used to refine structure: *SHELXTL*; molecular graphics: *SHELXTL*; software used to prepare material for publication: *SHELXTL* and *PLATON* (Spek, 2009 ▶).

## Supplementary Material

Crystal structure: contains datablock(s) global, I. DOI: 10.1107/S1600536811034994/hb5945sup1.cif

Structure factors: contains datablock(s) I. DOI: 10.1107/S1600536811034994/hb5945Isup2.hkl

Supplementary material file. DOI: 10.1107/S1600536811034994/hb5945Isup3.cml

Additional supplementary materials:  crystallographic information; 3D view; checkCIF report

## Figures and Tables

**Table 1:** Hydrogen-bond geometry (Å, °) *Cg*1, *Cg*3, *Cg*5, *Cg*6 and *Cg*7 are the centroids of the C1*A*–C6*A*, C8*A*–C13*A*, C1*B*–C6*B*, C1*B*/C6*B*–C8*B*/C13*B*–C14*B* and C8*B*–C13*B* rings, respectively.

*D*—H⋯*A*	*D*—H	H⋯*A*	*D*⋯*A*	*D*—H⋯*A*
O2*A*—H1*OA*⋯O1*A*	0.93 (2)	1.69 (2)	2.5459 (12)	152.2 (19)
O2*B*—H1*OB*⋯O1*B*	0.88 (2)	1.75 (2)	2.5725 (13)	154.2 (19)
C5*A*—H5*AA*⋯*Cg*5	0.93	2.84	3.6754 (13)	151
C7*A*—H7*AA*⋯*Cg*6	0.93	2.76	3.6440 (12)	158
C9*A*—H9*AA*⋯*Cg*7	0.93	2.73	3.6325 (12)	164
C9*B*—H9*BA*⋯*Cg*1^i^	0.93	2.76	3.4023 (12)	127
C23*B*—H23*B*⋯*Cg*3	0.93	2.91	3.7661 (11)	154

Symmetry code: (i) 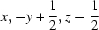.

**Fig. 1.**
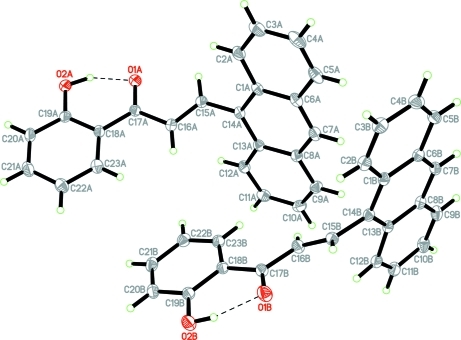
The molecular structure of the title compound, showing 50% probability displacement ellipsoids. O—H···O hydrogen bonds are shown as dashed lines.

**Fig. 2.**
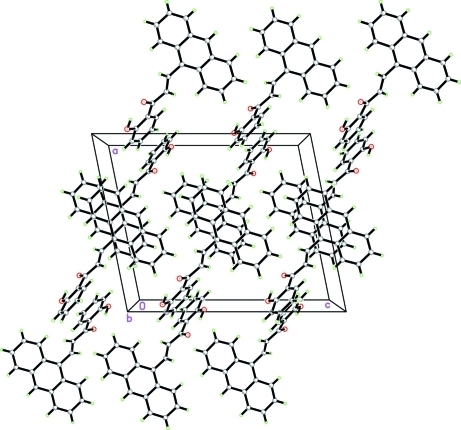
The crystal packing of the title compound viewed along the b axis.

## supplementary crystallographic information

### Comment

From our previous work, which revealed that a chalcone derivative containing the anthracene moiety displayed fluorescence (Joothamongkhon *et al.*, 2010), the title compound (I) was synthesized by changing the substituent group on the phenyl ring for comparison of their properties.   It was then discovered that a recent study of the same structure (Jasinski *et al.*, 2011) contained errors in the title, scheme and synthesis. It was found that (I) exhibits fluorescence with the maximum emission at 438 nm when excited at 380 nm in chloroform solution. In addition our experiment shows that (I) also exhibits tyrosinase inhibitory activity with % inhibition of 12.882±8.511 at the concentration 0.125 mg ml^-1^ when *L*-tyrosine was used as substrate.

The asymmetric unit of (I) contains two molecules, *A* and *B*, with the same configuration but with slight differences in bond lengths and angles. The molecule of (I) (Fig. 1) exists in an *E* configuration with respect to the C15═C16 double bond [1.3392 (15) Å in molecule *A* and 1.3370 (15) Å in molecule *B*] and the torsion angle C14–C15–C16–C17 = 177.64 (10)° in molecule *A* [-179.49 (10)° in molecule *B*]. The anthracene unit is essentially planar with the *r.m.s*. 0.0270 (1) Å for molecule *A* [0.0236 (1) Å for molecule *B*]. Atom O1 of the prop-2-en-1-one (C15–C17/O1) moiety is deviated from the propene plane with the torsion angle C15–C16–C17–O1 = 18.56 (16)° in molecule *A* [-17.28 (17)° in molecule *B*]. The total molecule is twisted as the dihedral angle between phenyl and anthracene rings is 73.70 (4)° and the mean through the pro-2-en-1-one unit makes the dihedral angles of 14.70 (7) and 61.46 (6)° with the phenyl and anthracene rings, respectively [the corresponding values are 73.18 (4), 11.04 (7) and 62.15 (6)° in molecule *B*]. Intramolecular O2A—H1OA···O1A and O2B—H1OB..O1B hydrogen bonds (Table 1) generate S(6) ring motifs (Bernstein *et al.*, 1995).

The bond distances are comparable with those in the related structure noted above (Joothamongkhon *et al.*, 2010).

In the crystal (Fig. 2), the molecules are arranged into sheets parallel to the *ac* plane and further stacked along the *b* axis by π–π interactions with the centroid···centroid distances: *Cg*_1_···*Cg*_2_^ii^ = 3.6421 (6) Å; *Cg*_1_···*Cg*_3_^ii^ = 3.6800 (7) Å; *Cg*_2_···*Cg*_2_^ii^ = 3.7607 (6) Å; *Cg*_4_···*Cg*_8_^iii^ = 3.6434 (7) Å and *Cg*_5_···*Cg*_6_^ii^ = 3.7084 (6) Å. The crystal structure is further stabilized by C—H···π interactions (Table 1); *Cg*_1_, *Cg*_2_, *Cg*_3_, *Cg*_4_, *Cg*_5_, *Cg*_6_, *Cg*_7_ and *Cg*_8_ are the centroids of C1A–C6A, C1A/C6A–C8A/C13A–C14A, C8A–C13A, C18A–C23A, C1B–C6B, C1B/C6B–C8B/C13B–C14B, C8B–C13B and C18B–C23B rings, respectively. C···O^iii^[3.2159 (15) Å] short contacts were also observed [symmetry code: (iii) -*x*, -1/2 + *y*, 1/2 - *z*].

### Experimental

The title compound was synthesized by the condensation of anthracene-9-carbaldehyde (2 mmol, 0.41 g) with 2-hydroxyacetophenone (2 mmol, 0.27 g) in ethanol (40 ml) in the presence of NaOH(aq) (10 ml, 40%). After stirring for 4 hr at room temperature, a yellow solid appeared and was then collected by filtration, washed with distilled water and dried in air. Yellow blocks of (I) were recrystalized from acetone by the slow evaporation of the solvent at room temperature after several days, Mp. 431–432 K.

### Refinement

Hydroxy H atoms were located in a difference maps and refined isotropically. The remaining H atoms were positioned geometrically and allowed to ride on their parent atoms, with d(C—H) = 0.93 Å for aromatic and CH. and the *U*_iso_ values were constrained to be 1.2*U*_eq_ of the carrier atoms. The highest residual electron density peak is located at 0.71 Å from C1A and the deepest hole is located at 0.43 Å from H1OA.

### Crystal data

**Table:**

C_23_H_16_O_2_	*F*(000) = 1360
*M**_r_* = 324.36	*D*_x_ = 1.341 Mg m^−^^3^
Monoclinic, *P*2_1_/*c*	Melting point = 431–432 K
Hall symbol: -P 2ybc	Mo *K*α radiation, λ = 0.71073 Å
*a* = 14.0843 (2) Å	Cell parameters from 9368 reflections
*b* = 13.7224 (2) Å	θ = 1.9–30.0°
*c* = 16.9615 (3) Å	µ = 0.09 mm^−^^1^
β = 101.411 (1)°	*T* = 100 K
*V* = 3213.36 (9) Å^3^	Block, yellow
*Z* = 8	0.50 × 0.39 × 0.37 mm

### Data collection

**Table:**

Bruker APEXII CCD diffractometer	9368 independent reflections
Radiation source: sealed tube	7868 reflections with *I* > 2σ(*I*)
graphite	*R*_int_ = 0.032
φ and ω scans	θ_max_ = 30.0°, θ_min_ = 1.9°
Absorption correction: multi-scan (*SADABS*; Bruker, 2005)	*h* = −19→19
*T*_min_ = 0.959, *T*_max_ = 0.969	*k* = −17→19
40230 measured reflections	*l* = −23→23

### Refinement

**Table:**

Refinement on *F*^2^	Primary atom site location: structure-invariant direct methods
Least-squares matrix: full	Secondary atom site location: difference Fourier map
*R*[*F*^2^ > 2σ(*F*^2^)] = 0.046	Hydrogen site location: inferred from neighbouring sites
*wR*(*F*^2^) = 0.132	H atoms treated by a mixture of independent and constrained refinement
*S* = 1.02	*w* = 1/[σ^2^(*F*_o_^2^) + (0.0739*P*)^2^ + 1.125*P*] where *P* = (*F*_o_^2^ + 2*F*_c_^2^)/3
9368 reflections	(Δ/σ)_max_ = 0.001
459 parameters	Δρ_max_ = 0.54 e Å^−^^3^
0 restraints	Δρ_min_ = −0.21 e Å^−^^3^

### Special details

**Table:**

Experimental. The crystal was placed in the cold stream of an Oxford Cryosystems Cobra open-flow nitrogen cryostat (Cosier & Glazer, 1986) operating at 120.0 (1) K.
Geometry. All e.s.d.'s (except the e.s.d. in the dihedral angle between two l.s. planes) are estimated using the full covariance matrix. The cell e.s.d.'s are taken into account individually in the estimation of e.s.d.'s in distances, angles and torsion angles; correlations between e.s.d.'s in cell parameters are only used when they are defined by crystal symmetry. An approximate (isotropic) treatment of cell e.s.d.'s is used for estimating e.s.d.'s involving l.s. planes.
Refinement. Refinement of *F*^2^ against ALL reflections. The weighted *R*-factor *wR* and goodness of fit *S* are based on *F*^2^, conventional *R*-factors *R* are based on *F*, with *F* set to zero for negative *F*^2^. The threshold expression of *F*^2^ > σ(*F*^2^) is used only for calculating *R*-factors(gt) *etc*. and is not relevant to the choice of reflections for refinement. *R*-factors based on *F*^2^ are statistically about twice as large as those based on *F*, and *R*- factors based on ALL data will be even larger.

### Fractional atomic coordinates and isotropic or equivalent isotropic displacement parameters (Å^2^)

**Table:**

	*x*	*y*	*z*	*U*_iso_*/*U*_eq_	
O1A	0.23244 (6)	0.20731 (6)	0.25499 (5)	0.02242 (17)	
O2A	0.12311 (6)	0.12104 (6)	0.13834 (5)	0.02449 (18)	
C1A	0.44611 (8)	0.49417 (8)	0.37297 (6)	0.0174 (2)	
C2A	0.50222 (8)	0.44622 (9)	0.32283 (6)	0.0227 (2)	
H2AA	0.4773	0.3914	0.2936	0.027*	
C3A	0.59212 (9)	0.48005 (11)	0.31737 (7)	0.0271 (3)	
H3AA	0.6279	0.4475	0.2850	0.033*	
C4A	0.63149 (9)	0.56412 (11)	0.36045 (7)	0.0279 (3)	
H4AA	0.6923	0.5867	0.3556	0.033*	
C5A	0.58036 (8)	0.61172 (9)	0.40878 (7)	0.0239 (2)	
H5AA	0.6069	0.6666	0.4371	0.029*	
C6A	0.48658 (8)	0.57869 (8)	0.41671 (6)	0.0185 (2)	
C7A	0.43416 (8)	0.62652 (8)	0.46690 (7)	0.0194 (2)	
H7AA	0.4608	0.6811	0.4955	0.023*	
C8A	0.34265 (8)	0.59435 (8)	0.47522 (6)	0.0173 (2)	
C9A	0.29196 (9)	0.64164 (8)	0.52973 (7)	0.0210 (2)	
H9AA	0.3197	0.6953	0.5590	0.025*	
C10A	0.20378 (9)	0.60920 (9)	0.53942 (7)	0.0231 (2)	
H10A	0.1713	0.6412	0.5746	0.028*	
C11A	0.16130 (8)	0.52659 (9)	0.49592 (7)	0.0217 (2)	
H11A	0.1012	0.5045	0.5032	0.026*	
C12A	0.20734 (8)	0.47903 (8)	0.44350 (6)	0.0188 (2)	
H12A	0.1782	0.4248	0.4159	0.023*	
C13A	0.29970 (8)	0.51123 (8)	0.43031 (6)	0.01584 (19)	
C14A	0.35199 (8)	0.46226 (8)	0.37893 (6)	0.01602 (19)	
C15A	0.31128 (8)	0.37739 (8)	0.33147 (6)	0.0184 (2)	
H15A	0.3487	0.3211	0.3358	0.022*	
C16A	0.22424 (8)	0.37492 (8)	0.28239 (6)	0.0199 (2)	
H16A	0.1840	0.4292	0.2782	0.024*	
C17A	0.19193 (8)	0.28682 (8)	0.23504 (6)	0.0179 (2)	
C18A	0.11383 (8)	0.29411 (8)	0.16329 (6)	0.0172 (2)	
C19A	0.08536 (8)	0.20943 (9)	0.11699 (6)	0.0193 (2)	
C20A	0.01483 (9)	0.21635 (10)	0.04625 (7)	0.0242 (2)	
H20A	−0.0024	0.1613	0.0147	0.029*	
C21A	−0.02897 (9)	0.30437 (10)	0.02334 (7)	0.0252 (2)	
H21A	−0.0761	0.3080	−0.0234	0.030*	
C22A	−0.00370 (9)	0.38831 (9)	0.06921 (7)	0.0240 (2)	
H22A	−0.0346	0.4472	0.0538	0.029*	
C23A	0.06780 (8)	0.38290 (9)	0.13788 (7)	0.0209 (2)	
H23A	0.0858	0.4390	0.1679	0.025*	
O1B	0.18383 (6)	0.94230 (6)	0.26687 (5)	0.02468 (18)	
O2B	0.03389 (7)	0.89265 (7)	0.16165 (5)	0.02348 (18)	
C1B	0.50277 (8)	0.87960 (7)	0.47232 (6)	0.01604 (19)	
C2B	0.53592 (8)	0.88359 (8)	0.39761 (6)	0.0194 (2)	
H2BA	0.4912	0.8925	0.3499	0.023*	
C3B	0.63166 (9)	0.87455 (9)	0.39523 (7)	0.0221 (2)	
H3BA	0.6512	0.8783	0.3461	0.026*	
C4B	0.70218 (9)	0.85953 (9)	0.46673 (7)	0.0223 (2)	
H4BA	0.7673	0.8534	0.4641	0.027*	
C5B	0.67406 (8)	0.85419 (8)	0.53925 (7)	0.0198 (2)	
H5BA	0.7203	0.8442	0.5859	0.024*	
C6B	0.57425 (8)	0.86375 (8)	0.54417 (6)	0.0166 (2)	
C7B	0.54489 (8)	0.85640 (8)	0.61815 (6)	0.0173 (2)	
H7BA	0.5912	0.8461	0.6647	0.021*	
C8B	0.44772 (8)	0.86415 (8)	0.62337 (6)	0.0175 (2)	
C9B	0.41872 (9)	0.85451 (9)	0.69944 (6)	0.0220 (2)	
H9BA	0.4652	0.8414	0.7453	0.026*	
C10B	0.32440 (9)	0.86416 (10)	0.70549 (7)	0.0253 (2)	
H10B	0.3065	0.8564	0.7550	0.030*	
C11B	0.25298 (9)	0.88619 (9)	0.63594 (7)	0.0233 (2)	
H11B	0.1888	0.8945	0.6407	0.028*	
C12B	0.27755 (8)	0.89535 (8)	0.56208 (7)	0.0196 (2)	
H12B	0.2298	0.9104	0.5175	0.024*	
C13B	0.37537 (8)	0.88220 (8)	0.55225 (6)	0.0165 (2)	
C14B	0.40387 (8)	0.88795 (7)	0.47662 (6)	0.01590 (19)	
C15B	0.33125 (8)	0.90127 (8)	0.40140 (6)	0.0180 (2)	
H15B	0.3405	0.9525	0.3679	0.022*	
C16B	0.25332 (8)	0.84486 (8)	0.37825 (6)	0.0189 (2)	
H16B	0.2422	0.7934	0.4110	0.023*	
C17B	0.18418 (8)	0.86241 (8)	0.30157 (6)	0.0181 (2)	
C18B	0.11590 (8)	0.78474 (8)	0.26706 (6)	0.0167 (2)	
C19B	0.04372 (8)	0.80431 (8)	0.19787 (6)	0.0184 (2)	
C20B	−0.02191 (8)	0.73166 (9)	0.16524 (6)	0.0215 (2)	
H20B	−0.0693	0.7450	0.1200	0.026*	
C21B	−0.01681 (8)	0.64008 (9)	0.19984 (7)	0.0229 (2)	
H21B	−0.0609	0.5922	0.1778	0.027*	
C22B	0.05418 (9)	0.61899 (9)	0.26782 (7)	0.0217 (2)	
H22B	0.0578	0.5572	0.2907	0.026*	
C23B	0.11898 (8)	0.69067 (8)	0.30083 (6)	0.0190 (2)	
H23B	0.1656	0.6765	0.3463	0.023*	
H1OA	0.1696 (15)	0.1326 (15)	0.1845 (13)	0.052 (6)*	
H1OB	0.0847 (15)	0.9246 (15)	0.1878 (12)	0.052 (6)*	

### Atomic displacement parameters (Å^2^)

**Table:**

	*U*^11^	*U*^22^	*U*^33^	*U*^12^	*U*^13^	*U*^23^
O1A	0.0194 (4)	0.0219 (4)	0.0243 (4)	0.0023 (3)	0.0002 (3)	−0.0037 (3)
O2A	0.0203 (4)	0.0243 (4)	0.0272 (4)	0.0028 (3)	0.0006 (3)	−0.0089 (3)
C1A	0.0145 (5)	0.0220 (5)	0.0147 (4)	−0.0008 (4)	0.0008 (3)	0.0034 (4)
C2A	0.0185 (5)	0.0334 (6)	0.0157 (4)	0.0012 (4)	0.0021 (4)	0.0007 (4)
C3A	0.0182 (5)	0.0450 (8)	0.0187 (5)	0.0036 (5)	0.0048 (4)	0.0056 (5)
C4A	0.0159 (5)	0.0417 (7)	0.0254 (5)	−0.0036 (5)	0.0026 (4)	0.0124 (5)
C5A	0.0177 (5)	0.0273 (6)	0.0247 (5)	−0.0061 (4)	−0.0005 (4)	0.0094 (4)
C6A	0.0166 (5)	0.0195 (5)	0.0181 (4)	−0.0027 (4)	0.0004 (4)	0.0062 (4)
C7A	0.0206 (5)	0.0150 (5)	0.0209 (5)	−0.0032 (4)	−0.0001 (4)	0.0028 (4)
C8A	0.0187 (5)	0.0149 (5)	0.0173 (4)	0.0000 (4)	0.0011 (4)	0.0024 (3)
C9A	0.0255 (6)	0.0155 (5)	0.0210 (5)	0.0025 (4)	0.0023 (4)	−0.0006 (4)
C10A	0.0257 (6)	0.0226 (6)	0.0216 (5)	0.0057 (4)	0.0065 (4)	0.0009 (4)
C11A	0.0182 (5)	0.0245 (6)	0.0232 (5)	0.0006 (4)	0.0062 (4)	0.0034 (4)
C12A	0.0171 (5)	0.0193 (5)	0.0199 (5)	−0.0025 (4)	0.0030 (4)	0.0013 (4)
C13A	0.0159 (5)	0.0155 (5)	0.0154 (4)	−0.0003 (4)	0.0015 (3)	0.0019 (3)
C14A	0.0148 (5)	0.0178 (5)	0.0148 (4)	−0.0014 (4)	0.0013 (3)	0.0010 (3)
C15A	0.0176 (5)	0.0193 (5)	0.0182 (5)	−0.0005 (4)	0.0034 (4)	−0.0013 (4)
C16A	0.0194 (5)	0.0200 (5)	0.0192 (5)	0.0001 (4)	0.0016 (4)	−0.0035 (4)
C17A	0.0142 (5)	0.0221 (5)	0.0175 (4)	−0.0013 (4)	0.0037 (4)	−0.0028 (4)
C18A	0.0139 (5)	0.0222 (5)	0.0156 (4)	−0.0021 (4)	0.0036 (3)	−0.0017 (4)
C19A	0.0148 (5)	0.0242 (5)	0.0197 (5)	−0.0007 (4)	0.0055 (4)	−0.0046 (4)
C20A	0.0194 (5)	0.0319 (6)	0.0201 (5)	−0.0018 (5)	0.0012 (4)	−0.0084 (4)
C21A	0.0207 (5)	0.0365 (7)	0.0172 (5)	−0.0010 (5)	0.0010 (4)	−0.0010 (4)
C22A	0.0230 (6)	0.0274 (6)	0.0207 (5)	0.0000 (5)	0.0024 (4)	0.0045 (4)
C23A	0.0216 (5)	0.0221 (5)	0.0188 (5)	−0.0030 (4)	0.0032 (4)	0.0010 (4)
O1B	0.0254 (4)	0.0208 (4)	0.0249 (4)	−0.0021 (3)	−0.0021 (3)	0.0047 (3)
O2B	0.0221 (4)	0.0248 (4)	0.0214 (4)	0.0002 (3)	−0.0009 (3)	0.0023 (3)
C1B	0.0177 (5)	0.0129 (5)	0.0173 (4)	−0.0021 (4)	0.0028 (4)	−0.0009 (3)
C2B	0.0223 (5)	0.0185 (5)	0.0173 (5)	−0.0008 (4)	0.0038 (4)	0.0005 (4)
C3B	0.0242 (6)	0.0221 (5)	0.0217 (5)	−0.0015 (4)	0.0088 (4)	−0.0001 (4)
C4B	0.0179 (5)	0.0230 (6)	0.0273 (5)	−0.0004 (4)	0.0074 (4)	−0.0004 (4)
C5B	0.0165 (5)	0.0194 (5)	0.0228 (5)	−0.0003 (4)	0.0023 (4)	−0.0002 (4)
C6B	0.0173 (5)	0.0136 (5)	0.0186 (5)	−0.0009 (4)	0.0027 (4)	−0.0010 (3)
C7B	0.0179 (5)	0.0162 (5)	0.0169 (4)	−0.0013 (4)	0.0011 (4)	−0.0011 (3)
C8B	0.0195 (5)	0.0159 (5)	0.0169 (4)	−0.0029 (4)	0.0033 (4)	−0.0027 (3)
C9B	0.0234 (6)	0.0260 (6)	0.0163 (5)	−0.0046 (4)	0.0031 (4)	−0.0032 (4)
C10B	0.0254 (6)	0.0324 (6)	0.0193 (5)	−0.0059 (5)	0.0078 (4)	−0.0065 (4)
C11B	0.0183 (5)	0.0278 (6)	0.0249 (5)	−0.0035 (4)	0.0067 (4)	−0.0079 (4)
C12B	0.0177 (5)	0.0189 (5)	0.0219 (5)	−0.0018 (4)	0.0031 (4)	−0.0042 (4)
C13B	0.0172 (5)	0.0139 (5)	0.0180 (4)	−0.0020 (4)	0.0026 (4)	−0.0028 (3)
C14B	0.0170 (5)	0.0129 (5)	0.0171 (4)	−0.0015 (4)	0.0018 (4)	−0.0006 (3)
C15B	0.0193 (5)	0.0171 (5)	0.0172 (4)	0.0005 (4)	0.0023 (4)	0.0003 (4)
C16B	0.0188 (5)	0.0186 (5)	0.0182 (5)	−0.0012 (4)	0.0010 (4)	0.0013 (4)
C17B	0.0162 (5)	0.0194 (5)	0.0183 (4)	−0.0002 (4)	0.0026 (4)	−0.0006 (4)
C18B	0.0144 (5)	0.0203 (5)	0.0155 (4)	0.0000 (4)	0.0035 (3)	−0.0020 (4)
C19B	0.0164 (5)	0.0231 (5)	0.0164 (4)	0.0019 (4)	0.0046 (4)	−0.0015 (4)
C20B	0.0163 (5)	0.0305 (6)	0.0173 (5)	−0.0011 (4)	0.0028 (4)	−0.0052 (4)
C21B	0.0191 (5)	0.0264 (6)	0.0237 (5)	−0.0049 (4)	0.0057 (4)	−0.0081 (4)
C22B	0.0212 (5)	0.0208 (5)	0.0241 (5)	−0.0019 (4)	0.0069 (4)	−0.0028 (4)
C23B	0.0172 (5)	0.0216 (5)	0.0184 (5)	0.0002 (4)	0.0042 (4)	−0.0014 (4)

### Geometric parameters (Å, °)

**Table:**

O1A—C17A	1.2466 (14)	O1B—C17B	1.2438 (14)
O2A—C19A	1.3449 (14)	O2B—C19B	1.3537 (14)
O2A—H1OA	0.93 (2)	O2B—H1OB	0.88 (2)
C1A—C14A	1.4186 (14)	C1B—C14B	1.4138 (15)
C1A—C2A	1.4306 (15)	C1B—C6B	1.4354 (14)
C1A—C6A	1.4329 (16)	C1B—C2B	1.4356 (14)
C2A—C3A	1.3688 (17)	C2B—C3B	1.3627 (16)
C2A—H2AA	0.9300	C2B—H2BA	0.9300
C3A—C4A	1.418 (2)	C3B—C4B	1.4224 (17)
C3A—H3AA	0.9300	C3B—H3BA	0.9300
C4A—C5A	1.3606 (19)	C4B—C5B	1.3668 (15)
C4A—H4AA	0.9300	C4B—H4BA	0.9300
C5A—C6A	1.4277 (15)	C5B—C6B	1.4307 (15)
C5A—H5AA	0.9300	C5B—H5BA	0.9300
C6A—C7A	1.3962 (16)	C6B—C7B	1.4003 (14)
C7A—C8A	1.3957 (15)	C7B—C8B	1.3930 (15)
C7A—H7AA	0.9300	C7B—H7BA	0.9300
C8A—C9A	1.4306 (15)	C8B—C9B	1.4344 (14)
C8A—C13A	1.4371 (15)	C8B—C13B	1.4383 (15)
C9A—C10A	1.3595 (17)	C9B—C10B	1.3590 (17)
C9A—H9AA	0.9300	C9B—H9BA	0.9300
C10A—C11A	1.4187 (17)	C10B—C11B	1.4231 (17)
C10A—H10A	0.9300	C10B—H10B	0.9300
C11A—C12A	1.3657 (15)	C11B—C12B	1.3699 (15)
C11A—H11A	0.9300	C11B—H11B	0.9300
C12A—C13A	1.4327 (15)	C12B—C13B	1.4315 (15)
C12A—H12A	0.9300	C12B—H12B	0.9300
C13A—C14A	1.4167 (14)	C13B—C14B	1.4200 (14)
C14A—C15A	1.4664 (15)	C14B—C15B	1.4802 (14)
C15A—C16A	1.3392 (15)	C15B—C16B	1.3370 (15)
C15A—H15A	0.9300	C15B—H15B	0.9300
C16A—C17A	1.4728 (15)	C16B—C17B	1.4821 (14)
C16A—H16A	0.9300	C16B—H16B	0.9300
C17A—C18A	1.4736 (14)	C17B—C18B	1.4767 (15)
C18A—C23A	1.4072 (16)	C18B—C23B	1.4095 (15)
C18A—C19A	1.4153 (15)	C18B—C19B	1.4179 (14)
C19A—C20A	1.4014 (15)	C19B—C20B	1.3972 (16)
C20A—C21A	1.3768 (18)	C20B—C21B	1.3828 (18)
C20A—H20A	0.9300	C20B—H20B	0.9300
C21A—C22A	1.3962 (17)	C21B—C22B	1.3989 (17)
C21A—H21A	0.9300	C21B—H21B	0.9300
C22A—C23A	1.3828 (16)	C22B—C23B	1.3827 (16)
C22A—H22A	0.9300	C22B—H22B	0.9300
C23A—H23A	0.9300	C23B—H23B	0.9300
			
C19A—O2A—H1OA	104.3 (13)	C19B—O2B—H1OB	102.6 (13)
C14A—C1A—C2A	122.30 (10)	C14B—C1B—C6B	120.07 (9)
C14A—C1A—C6A	119.63 (10)	C14B—C1B—C2B	122.56 (10)
C2A—C1A—C6A	118.04 (10)	C6B—C1B—C2B	117.34 (10)
C3A—C2A—C1A	120.75 (12)	C3B—C2B—C1B	121.28 (10)
C3A—C2A—H2AA	119.6	C3B—C2B—H2BA	119.4
C1A—C2A—H2AA	119.6	C1B—C2B—H2BA	119.4
C2A—C3A—C4A	120.91 (11)	C2B—C3B—C4B	121.07 (10)
C2A—C3A—H3AA	119.5	C2B—C3B—H3BA	119.5
C4A—C3A—H3AA	119.5	C4B—C3B—H3BA	119.5
C5A—C4A—C3A	120.09 (11)	C5B—C4B—C3B	119.82 (11)
C5A—C4A—H4AA	120.0	C5B—C4B—H4BA	120.1
C3A—C4A—H4AA	120.0	C3B—C4B—H4BA	120.1
C4A—C5A—C6A	120.94 (12)	C4B—C5B—C6B	120.71 (10)
C4A—C5A—H5AA	119.5	C4B—C5B—H5BA	119.6
C6A—C5A—H5AA	119.5	C6B—C5B—H5BA	119.6
C7A—C6A—C5A	121.26 (11)	C7B—C6B—C5B	120.89 (10)
C7A—C6A—C1A	119.48 (10)	C7B—C6B—C1B	119.33 (10)
C5A—C6A—C1A	119.26 (10)	C5B—C6B—C1B	119.77 (9)
C8A—C7A—C6A	121.55 (10)	C8B—C7B—C6B	121.30 (10)
C8A—C7A—H7AA	119.2	C8B—C7B—H7BA	119.4
C6A—C7A—H7AA	119.2	C6B—C7B—H7BA	119.4
C7A—C8A—C9A	120.72 (10)	C7B—C8B—C9B	120.55 (10)
C7A—C8A—C13A	119.85 (10)	C7B—C8B—C13B	120.12 (9)
C9A—C8A—C13A	119.41 (10)	C9B—C8B—C13B	119.33 (10)
C10A—C9A—C8A	120.95 (11)	C10B—C9B—C8B	121.02 (11)
C10A—C9A—H9AA	119.5	C10B—C9B—H9BA	119.5
C8A—C9A—H9AA	119.5	C8B—C9B—H9BA	119.5
C9A—C10A—C11A	120.00 (10)	C9B—C10B—C11B	119.96 (10)
C9A—C10A—H10A	120.0	C9B—C10B—H10B	120.0
C11A—C10A—H10A	120.0	C11B—C10B—H10B	120.0
C12A—C11A—C10A	120.98 (11)	C12B—C11B—C10B	120.83 (11)
C12A—C11A—H11A	119.5	C12B—C11B—H11B	119.6
C10A—C11A—H11A	119.5	C10B—C11B—H11B	119.6
C11A—C12A—C13A	121.14 (10)	C11B—C12B—C13B	121.21 (11)
C11A—C12A—H12A	119.4	C11B—C12B—H12B	119.4
C13A—C12A—H12A	119.4	C13B—C12B—H12B	119.4
C14A—C13A—C12A	123.25 (10)	C14B—C13B—C12B	123.29 (10)
C14A—C13A—C8A	119.15 (9)	C14B—C13B—C8B	119.16 (10)
C12A—C13A—C8A	117.51 (9)	C12B—C13B—C8B	117.54 (9)
C13A—C14A—C1A	120.29 (10)	C1B—C14B—C13B	119.97 (9)
C13A—C14A—C15A	121.33 (9)	C1B—C14B—C15B	119.04 (9)
C1A—C14A—C15A	118.38 (9)	C13B—C14B—C15B	120.98 (10)
C16A—C15A—C14A	124.87 (10)	C16B—C15B—C14B	124.67 (10)
C16A—C15A—H15A	117.6	C16B—C15B—H15B	117.7
C14A—C15A—H15A	117.6	C14B—C15B—H15B	117.7
C15A—C16A—C17A	120.39 (10)	C15B—C16B—C17B	121.49 (10)
C15A—C16A—H16A	119.8	C15B—C16B—H16B	119.3
C17A—C16A—H16A	119.8	C17B—C16B—H16B	119.3
O1A—C17A—C16A	119.67 (10)	O1B—C17B—C18B	120.49 (10)
O1A—C17A—C18A	120.69 (10)	O1B—C17B—C16B	119.92 (10)
C16A—C17A—C18A	119.62 (10)	C18B—C17B—C16B	119.59 (10)
C23A—C18A—C19A	118.57 (10)	C23B—C18B—C19B	117.98 (10)
C23A—C18A—C17A	122.35 (10)	C23B—C18B—C17B	122.32 (10)
C19A—C18A—C17A	119.07 (10)	C19B—C18B—C17B	119.70 (10)
O2A—C19A—C20A	117.85 (10)	O2B—C19B—C20B	117.32 (10)
O2A—C19A—C18A	122.52 (10)	O2B—C19B—C18B	122.50 (10)
C20A—C19A—C18A	119.63 (11)	C20B—C19B—C18B	120.17 (10)
C21A—C20A—C19A	120.22 (11)	C21B—C20B—C19B	120.36 (10)
C21A—C20A—H20A	119.9	C21B—C20B—H20B	119.8
C19A—C20A—H20A	119.9	C19B—C20B—H20B	119.8
C20A—C21A—C22A	121.06 (11)	C20B—C21B—C22B	120.44 (11)
C20A—C21A—H21A	119.5	C20B—C21B—H21B	119.8
C22A—C21A—H21A	119.5	C22B—C21B—H21B	119.8
C23A—C22A—C21A	119.21 (11)	C23B—C22B—C21B	119.57 (11)
C23A—C22A—H22A	120.4	C23B—C22B—H22B	120.2
C21A—C22A—H22A	120.4	C21B—C22B—H22B	120.2
C22A—C23A—C18A	121.27 (11)	C22B—C23B—C18B	121.48 (10)
C22A—C23A—H23A	119.4	C22B—C23B—H23B	119.3
C18A—C23A—H23A	119.4	C18B—C23B—H23B	119.3
			
C14A—C1A—C2A—C3A	178.79 (11)	C14B—C1B—C2B—C3B	−179.47 (10)
C6A—C1A—C2A—C3A	0.37 (16)	C6B—C1B—C2B—C3B	−1.22 (16)
C1A—C2A—C3A—C4A	−0.77 (18)	C1B—C2B—C3B—C4B	0.83 (18)
C2A—C3A—C4A—C5A	0.78 (18)	C2B—C3B—C4B—C5B	−0.13 (18)
C3A—C4A—C5A—C6A	−0.38 (17)	C3B—C4B—C5B—C6B	−0.13 (18)
C4A—C5A—C6A—C7A	179.27 (11)	C4B—C5B—C6B—C7B	178.72 (11)
C4A—C5A—C6A—C1A	−0.01 (16)	C4B—C5B—C6B—C1B	−0.31 (16)
C14A—C1A—C6A—C7A	2.27 (15)	C14B—C1B—C6B—C7B	0.20 (15)
C2A—C1A—C6A—C7A	−179.27 (10)	C2B—C1B—C6B—C7B	−178.10 (10)
C14A—C1A—C6A—C5A	−178.44 (10)	C14B—C1B—C6B—C5B	179.24 (10)
C2A—C1A—C6A—C5A	0.03 (15)	C2B—C1B—C6B—C5B	0.95 (15)
C5A—C6A—C7A—C8A	−179.69 (10)	C5B—C6B—C7B—C8B	−179.03 (10)
C1A—C6A—C7A—C8A	−0.41 (16)	C1B—C6B—C7B—C8B	0.01 (16)
C6A—C7A—C8A—C9A	177.12 (10)	C6B—C7B—C8B—C9B	178.75 (10)
C6A—C7A—C8A—C13A	−1.18 (16)	C6B—C7B—C8B—C13B	−1.40 (16)
C7A—C8A—C9A—C10A	−178.48 (10)	C7B—C8B—C9B—C10B	178.48 (11)
C13A—C8A—C9A—C10A	−0.17 (16)	C13B—C8B—C9B—C10B	−1.37 (17)
C8A—C9A—C10A—C11A	0.81 (17)	C8B—C9B—C10B—C11B	−1.32 (19)
C9A—C10A—C11A—C12A	−0.55 (17)	C9B—C10B—C11B—C12B	1.74 (19)
C10A—C11A—C12A—C13A	−0.37 (17)	C10B—C11B—C12B—C13B	0.62 (18)
C11A—C12A—C13A—C14A	177.54 (10)	C11B—C12B—C13B—C14B	177.98 (10)
C11A—C12A—C13A—C8A	0.98 (16)	C11B—C12B—C13B—C8B	−3.23 (16)
C7A—C8A—C13A—C14A	0.91 (15)	C7B—C8B—C13B—C14B	2.56 (15)
C9A—C8A—C13A—C14A	−177.41 (10)	C9B—C8B—C13B—C14B	−177.58 (10)
C7A—C8A—C13A—C12A	177.61 (10)	C7B—C8B—C13B—C12B	−176.28 (10)
C9A—C8A—C13A—C12A	−0.71 (15)	C9B—C8B—C13B—C12B	3.57 (15)
C12A—C13A—C14A—C1A	−175.56 (10)	C6B—C1B—C14B—C13B	1.00 (15)
C8A—C13A—C14A—C1A	0.95 (15)	C2B—C1B—C14B—C13B	179.20 (10)
C12A—C13A—C14A—C15A	3.81 (16)	C6B—C1B—C14B—C15B	−178.15 (9)
C8A—C13A—C14A—C15A	−179.68 (9)	C2B—C1B—C14B—C15B	0.05 (16)
C2A—C1A—C14A—C13A	179.07 (10)	C12B—C13B—C14B—C1B	176.41 (10)
C6A—C1A—C14A—C13A	−2.53 (15)	C8B—C13B—C14B—C1B	−2.36 (15)
C2A—C1A—C14A—C15A	−0.31 (15)	C12B—C13B—C14B—C15B	−4.45 (16)
C6A—C1A—C14A—C15A	178.08 (9)	C8B—C13B—C14B—C15B	176.78 (10)
C13A—C14A—C15A—C16A	52.54 (15)	C1B—C14B—C15B—C16B	125.91 (12)
C1A—C14A—C15A—C16A	−128.08 (12)	C13B—C14B—C15B—C16B	−53.23 (16)
C14A—C15A—C16A—C17A	177.64 (10)	C14B—C15B—C16B—C17B	−179.49 (10)
C15A—C16A—C17A—O1A	18.56 (16)	C15B—C16B—C17B—O1B	−17.28 (17)
C15A—C16A—C17A—C18A	−159.78 (10)	C15B—C16B—C17B—C18B	162.91 (10)
O1A—C17A—C18A—C23A	−178.62 (10)	O1B—C17B—C18B—C23B	174.78 (10)
C16A—C17A—C18A—C23A	−0.30 (15)	C16B—C17B—C18B—C23B	−5.41 (15)
O1A—C17A—C18A—C19A	−0.13 (15)	O1B—C17B—C18B—C19B	−5.71 (16)
C16A—C17A—C18A—C19A	178.20 (9)	C16B—C17B—C18B—C19B	174.10 (9)
C23A—C18A—C19A—O2A	−177.65 (10)	C23B—C18B—C19B—O2B	179.06 (9)
C17A—C18A—C19A—O2A	3.80 (15)	C17B—C18B—C19B—O2B	−0.47 (15)
C23A—C18A—C19A—C20A	1.96 (15)	C23B—C18B—C19B—C20B	0.22 (15)
C17A—C18A—C19A—C20A	−176.59 (10)	C17B—C18B—C19B—C20B	−179.31 (10)
O2A—C19A—C20A—C21A	177.39 (11)	O2B—C19B—C20B—C21B	−179.19 (10)
C18A—C19A—C20A—C21A	−2.24 (17)	C18B—C19B—C20B—C21B	−0.28 (16)
C19A—C20A—C21A—C22A	0.64 (18)	C19B—C20B—C21B—C22B	−0.11 (17)
C20A—C21A—C22A—C23A	1.23 (18)	C20B—C21B—C22B—C23B	0.56 (17)
C21A—C22A—C23A—C18A	−1.48 (17)	C21B—C22B—C23B—C18B	−0.63 (16)
C19A—C18A—C23A—C22A	−0.11 (16)	C19B—C18B—C23B—C22B	0.24 (15)
C17A—C18A—C23A—C22A	178.39 (10)	C17B—C18B—C23B—C22B	179.76 (10)

### Hydrogen-bond geometry (Å, °)

**Table:**

*Cg*1, *Cg*3, *Cg*5, *Cg*6 and *Cg*7 are the centroids of the C1A–C6A, C8A–C13A, C1B–C6B, C1B/C6B–C8B/C13B–C14B and C8B–C13B rings, respectively.

**Table:**

*D*—H···*A*	*D*—H	H···*A*	*D*···*A*	*D*—H···*A*
O2A—H1OA···O1A	0.93 (2)	1.69 (2)	2.5459 (12)	152.2 (19)
O2B—H1OB···O1B	0.88 (2)	1.75 (2)	2.5725 (13)	154.2 (19)
C5A—H5AA···Cg5	0.93	2.84	3.6754 (13)	151
C7A—H7AA···Cg6	0.93	2.76	3.6440 (12)	158
C9A—H9AA···Cg7	0.93	2.73	3.6325 (12)	164
C9B—H9BA···Cg1^i^	0.93	2.76	3.4023 (12)	127
C23B—H23B···Cg3	0.93	2.91	3.7661 (11)	154

Symmetry codes:  (i) *x*, −*y*+1/2, *z*−1/2.
